# Occlusal effects on text reading: an eye-tracker study

**DOI:** 10.3389/fnsys.2024.1409251

**Published:** 2024-08-15

**Authors:** Maria Paola Tramonti Fantozzi, Vincenzo De Cicco, Andrea Bazzani, Enrico Cataldo, Luca Bruschini, Davide De Cicco, Paola d’Ascanio, Ugo Faraguna, Diego Manzoni

**Affiliations:** ^1^Department of Translational Research and of New Surgical and Medical Technologies, University of Pisa, Pisa, Italy; ^2^Institute of Management, Scuola Superiore Sant'Anna, Pisa, Italy; ^3^Department of Physics, University of Pisa, Pisa, Italy; ^4^Department of Surgical, Medical and Molecular Pathology and Critical Care Medicine, University of Pisa, Pisa, Italy; ^5^Maxillofacial Surgery Unit, Italian Stomatology Institute, Milan, Italy; ^6^Department of Developmental Neuroscience, IRCCS Fondazione Stella Maris, Pisa, Italy

**Keywords:** occlusal correction, EMG asymmetry, Locus Coeruleus, text reading, eye movements

## Abstract

**Introduction:**

Asymmetric electromyographic (EMG) activity during teeth clenching has been linked to cognitive impairment, as evaluated by the Spinnler-Tognoni matrices test, and to asymmetric pupil size (anisocoria). Anisocoria indicates an asymmetric Locus Coeruleus activity, leading to an asymmetric hemispheric excitability worsening cognitive performance. Bite splint wearing corrects EMG asymmetry, reduces anisocoria and improves cognitive performance. This study explores the possible effect of EMG asymmetry on oculomotor behavior during text reading.

**Methods:**

In subjects showing different degrees of EMG asymmetry during clenching, the number and duration of fixation periods during a reading task, performed under two different occlusal conditions were analyzed. The first lecture was executed with a dental impression (imprint) interposed between the dental arches (corrected condition) and the second one with the arches in direct contact (habitual condition), without clenching effort. The imprint reduced the EMG asymmetries during clenching.

**Results:**

In both occlusal conditions, total reading time correlated with duration of fixations, but not with their number. An inverse relation was observed between the number of fixations and their duration across individuals. Fixation frequency and duration were positively and negatively correlated with the amplitude of EMG asymmetry, respectively. Differently, total reading time was not related to the EMG asymmetry. When switching from the corrected to the habitual condition, an increase in the number of fixations and a reduction in their duration was observed, while total reading time could be either increased or decreased. An increased fixation frequency was observed in most of the subjects, while a reduced duration only among individuals with shorter reading times in habitual condition.

**Discussion:**

In the habitual condition, EMG asymmetry influences reading patterns (more saccades/shorter fixations, less saccades/longer fixations) in our sample. The changes in text reading behavior elicited by occlusal correction can be explained by assuming that occlusal disharmony negatively interferes with the reading task by increasing the number of saccades necessary for text scanning. This finding may also indicate an increased difficulty in processing of visual information. The potential involvement of trigeminal pathways in the relation between occlusal factors and oculomotor control is discussed.

## 1 Introduction

The asymmetry of hemispheric activity may induce a cognitive impairment. Research on brain injuries shows that a unilateral parietal lesion leads to cognitive visuo-spatial impairment (unilateral neglect), which is not observed when both sides of the brain are symmetrically injured (Lomber and Payne, 1996), suggesting that the disability arises by the inter-hemispheric imbalance, rather than the size of the damaged area. In humans, unilateral neglect may be greatly reduced by (1) depressing the activity of the undamaged hemisphere (Koch et al., 2012; Andres et al., 2020) or (2) stimulating the vestibular and neck proprioceptive afferents to the damaged hemisphere (Kerkhoff, 2001). These findings suggest that brain’s capability to represent space depends on maintaining a proper balance of activity between specific brain regions and peripheral afferents. According to De Cicco et al. (2017) trigeminal sensorimotor asymmetries can also impair cognitive functions in subjects with otherwise intact brains. Specifically, an asymmetry in the masseter electromyographic (EMG) activity during clenching was found to negatively affect cognitive visuo-spatial performance, as measured using the Spinnler-Tognoni matrices test (Spinnler and Tognoni, 1987), performed without occlusal effort. Moreover, this sensorimotor trigeminal unbalance diminished the positive effect of chewing on cognitive visuo-spatial performance (Tramonti Fantozzi et al., 2021b). The use of occlusal correction, either through an orthotic device or an implant prosthesis, reduced the trigeminal asymmetry and improved visuo-spatial performance (De Cicco et al., 2014, 2016; Tramonti Fantozzi et al., 2021a,c,d), suggesting a causal relationship between trigeminal asymmetries and cognitive impairment. It is possible that the proprioceptive trigeminal asymmetry introduces an asymmetry within the spatial representation of one’s body. Such an unbalanced representation could disturb cognitive processes. Interestingly, in these studies, the EMG asymmetry observed during clenching was strongly and positively correlated with an asymmetry in pupil size (anisocoria) evaluated without any occlusal effort (De Cicco et al., 2016; Tramonti Fantozzi et al., 2021a,c, 2023). Since pupil size is considered a proxy of the activity of noradrenergic Locus Coeruleus (LC) neurons (Silvetti et al., 2013; Murphy et al., 2014; Hoffing and Seitz, 2015; Kihara et al., 2015; Joshi et al., 2016; Reimer et al., 2016; Einhäuser, 2017; Mathôt, 2018; Joshi and Gold, 2020), the anisocoria could arise from an asymmetric LC discharge. This asymmetry may lead to an asymmetric body representation or else to an imbalance in the hemispheric activity. In both instances, a detrimental effects on cognitive functions would occurr, mirroring the phenomenon described by Lomber and Payne (1996). So, whatever the mechanism could be (altered body representation, hemispheric unbalance), the LC asymmetry may be the link between the trigeminal asymmetry and to the cognitive deficits. Notably, correcting occlusal issues not only reduced the trigeminal asymmetry but also the associated anisocoria (De Cicco et al., 2016; Tramonti Fantozzi et al., 2021a,c, 2023).

The main goal of the present investigation was to verify whether, in a population of adult subjects showing an asymmetric masseter EMG activity during clenching, the presence of trigeminal asymmetry elicits specific changes in the oculomotor performance evaluated during a reading. The number of saccades and the duration of fixations were evaluated using an eye-tracker. Each subject performed the reading task under two different occlusal conditions: with the dental arches in direct contact (habitual condition) and with a dental impression (imprint) interposed between them (corrected condition). The imprint used to correct the occlusion was designed to eliminate or minimize the EMG asymmetry during clenching. According to our hypothesis, we expected that oculomotor impairments would be present when the reading task was performed without imprint.

## 2 Materials and methods

### 2.1 Subjects

Experiments were carried out in 40 subjects (age: 38.38 ± 10.05 years; females (*n* = 17): 36.00 ± 9.82 years; males (*n* = 23): 40.13 ± 10.06 years), who signed a written informed consent. Considering the side with higher EMG activity as the hypertonic side and the side with lower EGM activity as the hypotonic side, subjects showed a baseline EMG asymmetry greater than 20%. The asymmetry was evaluated as follow:


$$\frac{200\left(EMG\;\mathrm{hypertonic}\;\mathrm{side}-EMG\;\mathrm{hypotonic}\;\mathrm{side}\right)}{\left(EMG\;\mathrm{hypertonic}\;\mathrm{side}+EMG\;\mathrm{hypotonic}\;\mathrm{side}\right)}$$

All subjects were native Italian speakers and unaffected by neurological or psychiatric disorders. The study was conducted in accordance with the Declaration of Helsinki and the experimental protocol was approved by the Ethical Committee of the Pisa University (endorsement 39/2022).

### 2.2 Masseter electromyographic activity evaluation

During clenching, right and left masseter EMG activities were recorded by a K6-I MyoTronics system (sample rate: 720 Hz, cut-off frequency: 15 Hz, notch filter: 50 Hz) using Duotrode Ag/AgCl electrodes (inter-electrode distance: 19.5 mm, MyoTronics, Seattle, WA, USA), placed as described by De Cicco et al. (2014). Automatically filtered EMG traces were fully rectified, and the values of burst duration and average EMG activity were computed. The clenching effort (2–4 s) began and ended upon experimenter’s request.

### 2.3 Occlusal correction: dental impression manufacturing

Subjects received 15 min of transcutaneous electrical nerve stimulation (TENS) of trigeminal motor branches (Nnoaham and Kumbang, 2008), using four couples of electrodes, positioned on both sides at the level of the incisura sigmoidea and the submental region. Biphasic (cathodal/anodal) current pulses (0.54 ms duration, 21–25 mA intensity), delivered by two I.A.C.E.R. stimulators (Martellago, Venice, Italy), induced repeated contractions of the masseters and mandible depressor muscles. The stimuli for the left and the right sides were adjusted to achieve symmetric muscle activation, with the frequency set at 40and 0.618 Hz for mandible depressor and elevator muscles, respectively. These patterns led to an alternating contraction of the masseters and a tonic contraction of depressor muscles, resulting in small amplitude mandibular movements (1 mm). After TENS, the mandibular resting posture was lower. A dental impression was then obtained by placing a self-hardening material between the dental arches. When the subject wore this dental impression during a clenching effort, the EMG asymmetry was greatly reduced or abolished.

### 2.4 Oculomotor assessment

After creating the dental impressions (imprint), all subjects participated in two consecutive reading sessions to assess their oculomotor performance. In the first session, the subject wore the dental impression (corrected condition), while in the second session, their dental arches were in direct contact (habitual condition). It has been observed that simply repeating the reading task enhance reading ease, leading to shorter total reading time and reductions in both the frequency and the duration of fixation periods (Hyönä and Niemi, 1990; Raney and Rayner, 1995; Raney et al., 2000). Therefore, we chose to begin the experiment under the occlusal condition that, based on our hypothesis, was supposed to result in the best reading performance. In this way, the lack of improvement or a worsening of the reading performance in the second test could have been attributed to the effects of dental impression removal.

#### 2.4.1 Apparatus

Gaze data were collected at 50 Hz using a 1,750 Tobii eye-tracker system (Tobii Technologies, Stockholm, Sweden). The instrument detects gaze position through a corneal reflection technique and is endowed with a 17” TFT monitor (1.280 × 1,024 resolution). The subject is illuminated by five near-infrared diodes placed above (three) and below (two) the screen. An image of the subject is obtained by an infrared light sensitive camera placed below the screen, between the bottom diodes. The subjects seated in comfortable chair at 60 cm from the screen, that was placed on a desk. The test was performed in an experimental room, under artificial lightening, with closed shutters. During the entire duration of the experiment, the context was never modified, and screen’s luminance was maintained constant. At the start of the reading session, a 16 points calibration of the eye-tracker was done for each subject.

#### 2.4.2 Stimuli

During the reading session, subjects were instructed to silently read a specific text displayed on the monitor. The text was in Italian language and consisted of 29 lines, each with an average of 8 words per line. Font size, font type and text content were kept consistent across conditions for all the subjects. Since no time constraints were imposed, participants were free to modulate their reading speed.

#### 2.4.3 Oculomotor behavior during the reading task

Fixations and saccades were directly identified by the eye-tracker. For each subject, the total number of fixations, the frequency of fixations per unit time (s), and their average duration of fixations (fixation duration) were computed. The number of fixations corresponds to that of saccadic movements performed by the subjects to scan the text. Moreover, the total reading time was recorded.

### 2.5 Statistical analysis

No outlier rejection was applied to the population analyzed. Oculomotor data were preliminary tested for distribution normality by the Kolmogorov–Smirnov test and by the Shapiro–Wilk test. The results of this screening have been shown in Table 1. Since the more demanding test (Shapiro–Wilk) indicated a significant deviation from normality (Table 1A), a Log10 transform was applied to the data. The transformation gave rise to a normal distribution for all the oculomotor parameters analyzed (Table 1B). Data were represented as mean ± standard deviation (SD). Between condition comparisons were performed by paired *t*-test. Correlations between variables were investigated by linear regression analysis. In addition to these classic parametric statistics, more robust tests less biased by data distribution were performed for both regression analysis (rank Spearman, Gamma and Kendall Tau correlation) and between-condition comparisons (Wilcoxon test). Most of statistical analyses were performed with Statistical Package for Social Sciences (SPSS, version 20). For non-parametric correlations the software STATISTICA (version 6.0) was utilized. The level of statistical significance was set at *p* < 0.05.

**Table 1:** Results of the normality test performed (Kolmogorov–Smirnov and Shapiro–Wilk) for the different ocular metrics recorded in the two occlusal conditions.

		A. Original data		B. Log transformation	
	Condition	Kolmogorov–Smirnov test (*p*)	Shapiro–Wilk test (*p*)	Kolmogorov–Smirnov test (*p*)	Shapiro–Wilk test (*p*)
Fixation number (Nos.)	Corrected	0.219	**0.028**	0.680	0.460
	Habitual	0.076	**<0.0005**	0.511	0.100
Fixation number per sec (Nos./s)	Corrected	0.374	**0.002**	0.881	0.184
	Habitual	0.094	**<0.0005**	0.812	0.638
Fixation duration (ms)	Corrected	0.105	**<0.0005**	0.881	0.749
	Habitual	0.199	**<0.0005**	0.812	0.638
Total reading time (ms)	Corrected	0.423	**<0.0005**	0.778	0.749
	Habitual	0.373	**<0.0005**	0.545	0.464

Bold *p* values denote significant deviation of the relative distributions from normality.

## 3 Results

### 3.1 Effects of occlusal correction on EMG activity

**Figure 1:**
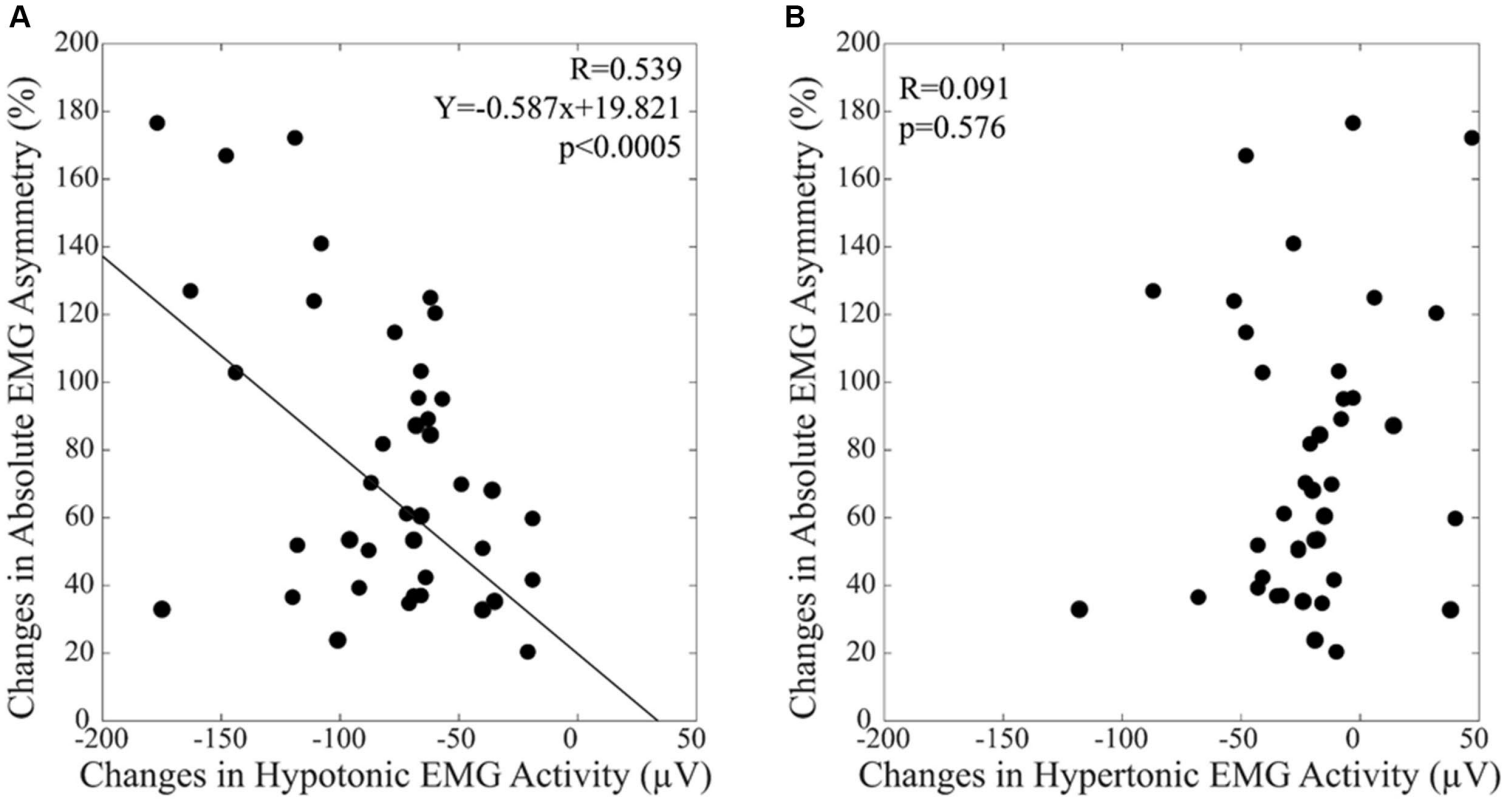
The differences in the absolute value of EMG asymmetry between by the habitual and the corrected conditions are plotted as a function of the EMG activity changes observed on the hypotonic **(A)** and hypertonic **(B)** side. A: The continuous line corresponds to the regression line for all the plotted points, the relative equation is displayed in the corresponding panel.

In the habitual condition, the EMG activity recorded on the hypotonic and hypertonic side corresponded to 61.9 ± 40.0 μV and to 126.0 ± 43.0 μV, respectively (*t*-test: *p* < 0.0005). Following occlusal correction, these EMG values were rebalanced (hypotonic: 143.0 ± 55.4 μV, hypertonic: 147.2 ± 51.0 μV, *t*-test: *p* = 0.090). The increase in EMG activity was significant for both sides (*t*-test: *p* < 0.0005). These modifications led to a significant reduction of the absolute value of EMG asymmetry in the corrected condition (from 76.8 ± 42.4% to 12.0 ± 9.8%, *t*-test: *p* < 0.0005). All these findings were confirmed by the Wilcoxon test. Basal values of EMG asymmetry and their reduction after occlusal correction were not correlated with subjects’ age. Linear regression analysis indicated that the (habitual - corrected) changes in EMG asymmetry were significantly correlated with those observed in the EMG activity recorded on the hypotonic side (Figure 1A). This finding was confirmed by non-parametric models of correlation (see Methods). Differently, no correlation was observed with the EMG changes recorded on the hypertonic side (Figure 1B).

### 3.2 Effects of changing occlusal condition on oculomotor measures and total reading time

**Figure 2:**
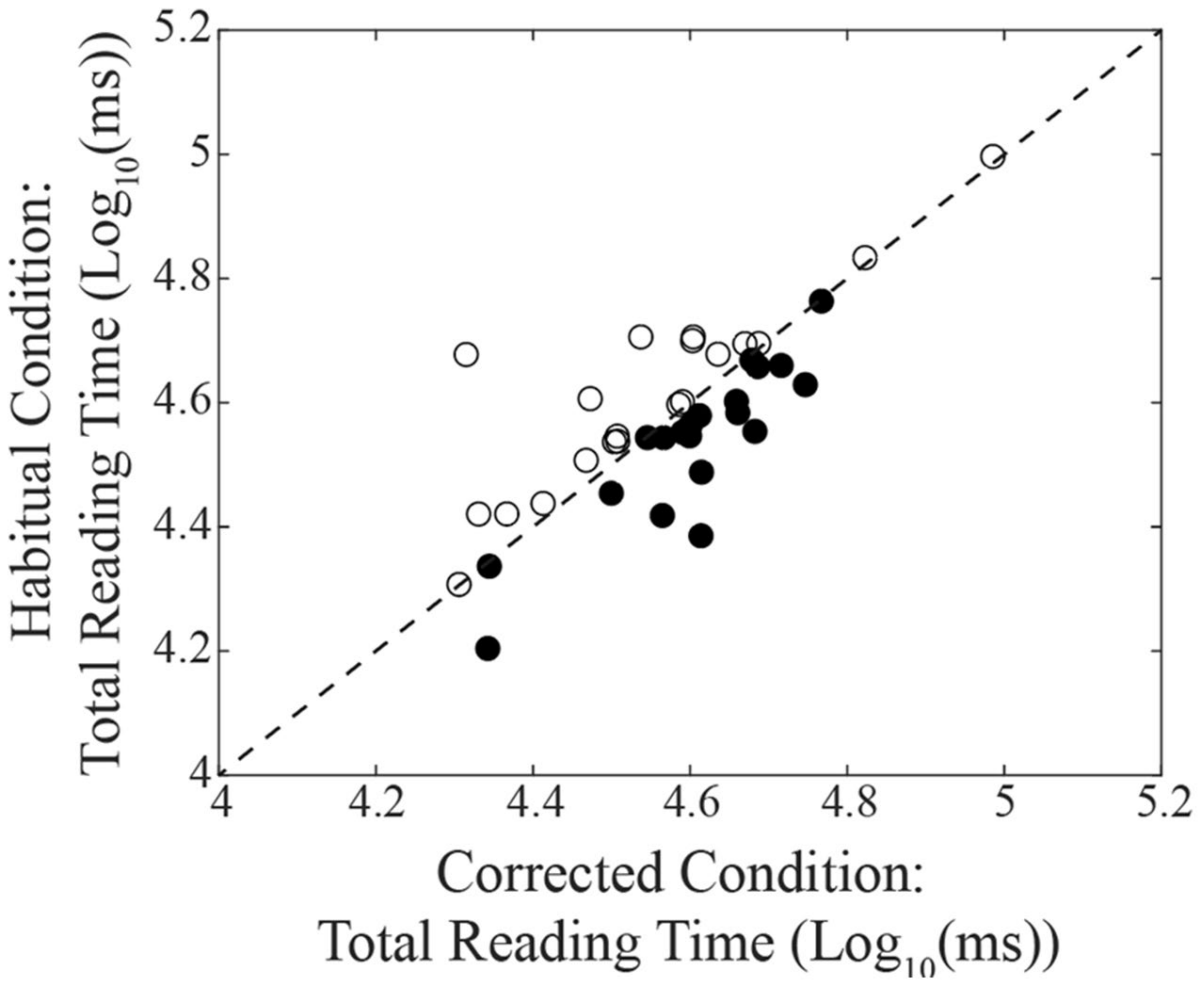
Relation between the total reading times observed in habitual and in corrected conditions. The dashed line represents equal values for the two conditions. Dots and circles represent subjects showing a decrease and an increase of their reading time, respectively, when changing from the corrected to the habitual condition.

**Figure 3:**
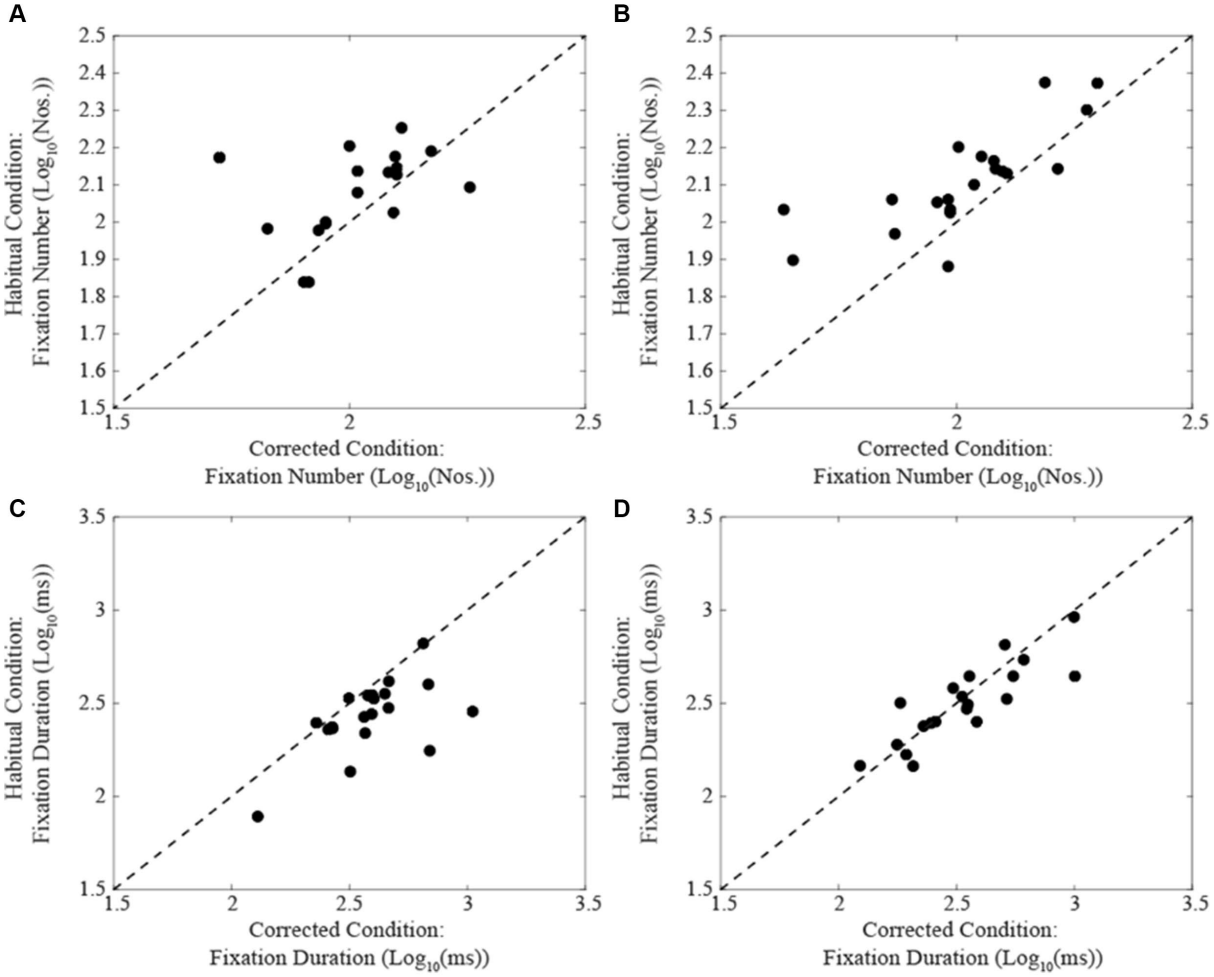
Comparison of fixation number **(A,B)** and duration values **(C,D)** obtained in habitual (ordinates) and corrected condition (abscissas) in subjects showing a decrease **(A,C)** or an increase **(B,D)** in their total reading time when switching from the corrected to the habitual condition. The dashed lines represent equal values for the two conditions.

A summary of the different oculomotor metrics recorded under corrected and habitual condition is displayed in Table 2. The corrected condition was associated with a lower fixation number compared to the habitual one. The same held true also for the number of fixations occurring per time unit. On the contrary, fixation duration was higher in corrected than in the habitual condition. Changing from the corrected to the habitual condition, no significant difference was observed in the total reading time. However, as shown in Figure 2, individual subjects could either show increases (circles) or decreases (dots) in this parameter. We will refer to these groups of subjects as Habitual Slower (HS) and Habitual Faster (HF), respectively. It is of interest that the increase in number of fixations characterized both HF (corrected: 2.02 ± 0.16 Log_10_(Nos.), habitual: 2.11 ± 0.16 Log_10_(Nos.), *t*-test: *p* = 0.019, Figure 3A) and HS (corrected: 2.02 ± 0.17 Log_10_(Nos.), habitual: 2.11 ± 0.13 Log_10_(Nos.), *t*-test: *p* = 0.001, Figure 3B) subjects. At variance, the decrease in fixation duration when passing from corrected to habitual condition was found among HF (corrected: 2.59 ± 0.20 Log_10_(ms); habitual: 2.43 ± 0.19 Log_10_(ms), *t*-test: *p* < 0.0005, Figure 3C) but not among HS subjects [corrected: 2.53 ± 0.24 Log_10_(ms); habitual: 2.50 ± 0.21 Log_10_(ms), *t*-test: *p* = 0.310, Figure 3D]. Nearly identical results were observed when data were compared by the Wilcoxon test. No differences were found between HF and HS subjects in terms of age, EMG asymmetry, oculomotor parameters and reading times evaluated in both corrected and habitual conditions.

**Table 2:** Mean ± SD values of oculomotor parameters evaluated in the corrected and habitual condition, compared by paired *t*-test and Wilcoxon test.

	A. Corrected condition	B. Habitual condition	A vs. B paired *t*-test (*p*)	A vs. B Wilcoxon test (*p*)
Fixation number [Log10(Nos.)]	2.02 ± 0.17	2.11 ± 0.14	<0.0005	<0.0005
Fixation number per sec [Log10(Nos./s)]	0.44 ± 0.22	0.54 ± 0,20	0.001	0.001
Fixation duration [Log10(ms)]	2.56 ± 0.22	2.46 ± 0.20	0.001	0.001
Total Reading time [Log10(ms)]	4.58 ± 0.14	4.57 ± 0.15	NS	NS

### 3.3 Correlations between oculomotor measures and EMG asymmetry

**Figure 4:**
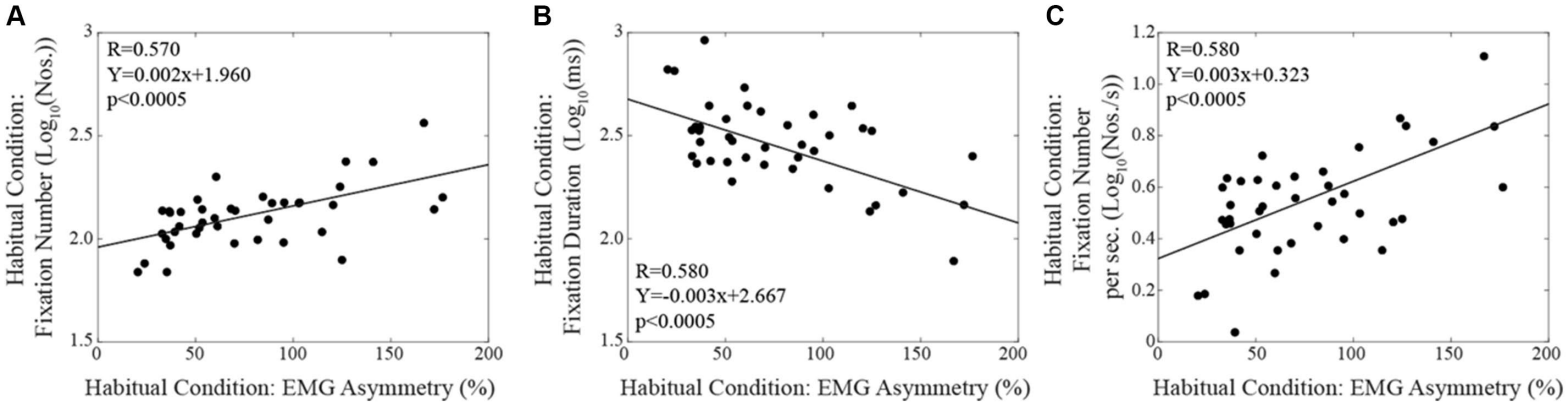
Habitual condition: correlations between the absolute value of EMG asymmetry and the fixation numbers **(A)**, the fixation duration **(B)** and the fixation number per time unit **(C)**. Continuous lines correspond to the regression lines for all the plotted points, the equations of which are displayed in the corresponding panel.

**Figure 5:**
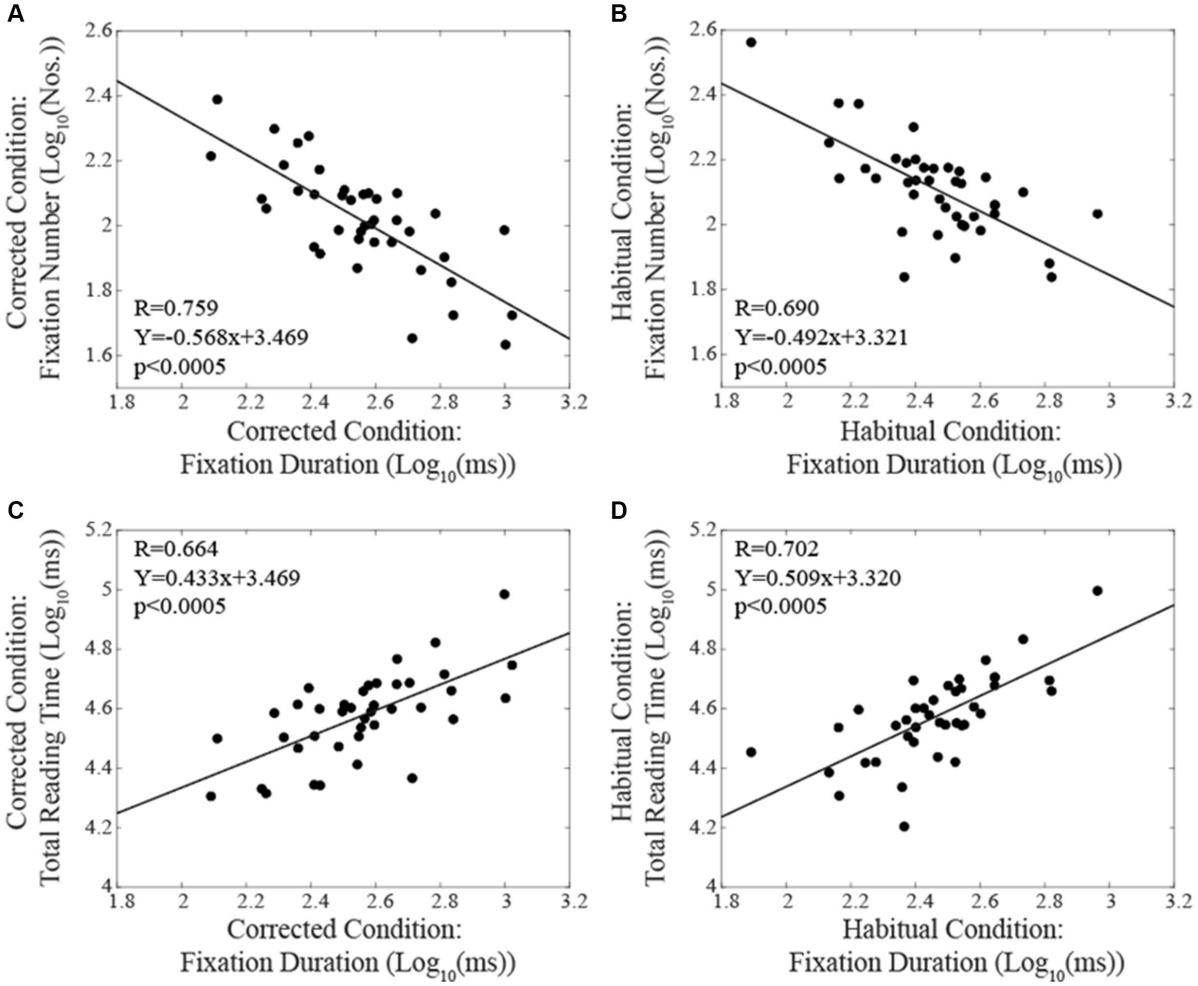
Correlations between oculomotor performance parameters and total reading time. The upper row represents the relation between number and duration of fixations in corrected **(A)** and habitual **(B)** conditions. The lower row displays the relation between total reading time and fixation duration in corrected **(C)** and habitual **(D)** conditions. Continuous lines correspond to the regression lines for all the plotted points, whose equations are displayed in the corresponding panel.

When subjects were tested in the corrected condition, linear regression analysis did not show any significant correlation between total reading time/oculomotor measurements and the corresponding level of EMG asymmetry. Differently, in the habitual condition, the number of fixations (Figure 4A) and their duration (Figure 4B) were positively and negatively correlated with the EMG asymmetry, respectively. A positive correlation with EMG asymmetry was also found when the number of fixations was divided by the total reading time (number of fixations per second, Figure 4C). Differently, no significant correlation was observed between the total reading time and the EMG asymmetry (R = 0.212, *p* = 0.189). All these correlations were confirmed by applying non-parametric models. As shown in Figure 5, in both the corrected (Figure 5A) and the habitual (Figure 5B) conditions, fixation number and fixation duration were strongly (and negatively) correlated. While the total reading time was independent upon the fixation frequency, it was positively correlated with the fixation duration in both the corrected (Figure 5C) and the habitual (Figure 5D) conditions. These finding were confirmed by non-parametric regression models.

### 3.4 Changes in oculomotor measures and EMG activity/asymmetry elicited by occlusal correction

**Figure 6:**
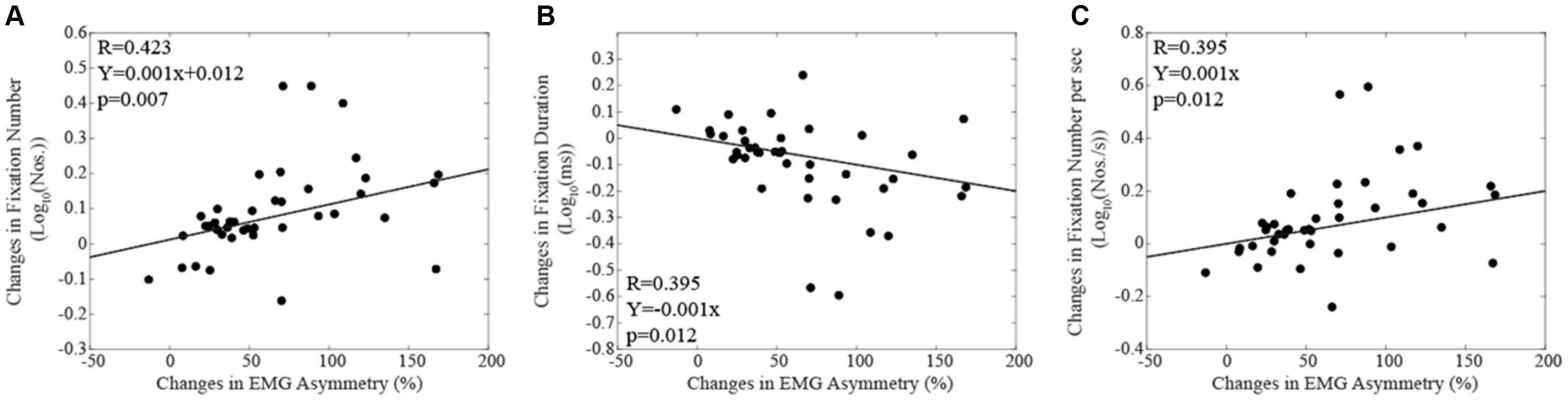
The changes in fixation number **(A)**, in fixation duration **(B)** and in fixation number per sec **(C)** elicited by switching from the corrected to the habitual condition are plotted as a function of the relative change in EMG asymmetry. Continuous lines correspond to the regression lines for all the plotted points, the equations of which are displayed in the corresponding panel.

The differences in the number of fixations (Figure 6A), in fixation duration (Figure 6B) and in the frequency of fixations per second (Figure 6C) observed by switching from the corrected to the habitual condition (Log habitual - Log corrected) were influenced by the relative changes in (habitual-corrected) EMG asymmetry, as indicated by linear regression analysis. This observation was confirmed by non-parametric correlations.

## 4 Discussion

In this study, we investigated oculomotor behaviour during a reading task in subjects under two different occlusal conditions: corrected and habitual. For all participants, the habitual condition was associated to an asymmetrical development of masseter EMG activity during clenching. Previous findings showed that, in this condition, cognitive visuo-spatial performance and task-induced mydriasis are significantly reduced (De Cicco et al., 2014, 2016; Tramonti Fantozzi et al., 2021a,c,d).

Reading fluently requires coordinated perceptual, cognitive, and motor skills (Reichle and Reingold, 2013). The phenomenon of fixation, closely related to visual functions, is a brief period (Rayner, 1998) during which the reader extracts and processes information associated with the word currently fixed (Liversedge and Findlay, 2000; Degno and Liversedge, 2020). The duration and/or location of fixations can be influenced by several factors, including words frequency and predictability (Rayner and Duffy, 1986; Rayner and Well, 1996; Rayner et al., 2006; Schotter et al., 2012). Brain regions actively involved in modulating fixations are the reticular formation (RF), the cerebellum, the superior colliculus, the parietal and the frontal cortex (Krauzlis et al., 2017). The neuronal mechanism responsible for regulating ocular fixation during reading involves several phases: (1) the transmission of the signal from the retina to the visual cortex, (2) the visual representation of the fixed word, (3) lexical processing of the word, and (4) the programming of saccadic movements (Reichle and Reingold, 2013).

### 4.1 Limitations of the study

The main limitation of the present study is the lack of a reversal in the order of the two occlusal conditions (corrected, habitual) under which the reading task was performed. The lack of reversal poses the problem of how to reasonably dissect the effects of the condition from those of repeating twice the same test (the corrected condition was tested before the habitual condition). In this respect, the data present in the literature allow to draw some conclusion despite the methodological flaw. Indeed, there is a large body of evidence that the second reading of a text results in a total reading time reduction and in a decrease in both the frequency and the duration of fixation periods (Hyönä and Niemi, 1990; Raney and Rayner, 1995; Raney et al., 2000). Reading difficulties, such as dyslexia, are typically characterized by the opposite pattern, i.e., an increase in both the number and the duration of fixation and an extension of the total reading time (Hutzler and Wimmer, 2004). This pattern has been observed also in patients with macular degeneration (Ktistakis et al., 2023), Alzheimer’s disease (Lueck et al., 2000; Fernández et al., 2013, 2015) and aphasia-based alexia (Hux et al., 2024). So, in the present study, the lack of an improvement or a worsening of oculomotor parameters during the second session suggests a negative effect of the habitual condition on text reading. Indeed, according to our hypothesis, the balanced (corrected) occlusal condition tested first should have been that associated to a better oculomotor control. Another point that allows to distinguish the effect of occlusal condition from those of task repetition is the fact that, in the former case, the changes observed in oculomotor behavior should be correlated with the associated changes in EMG asymmetry. This was indeed observed in the present study, as shown in Figure 6.

Finally, another limitation of the study was the lack of measuring the saccade amplitudes, whose acquisition would have allowed a better understanding of the changes in oculomotor behavior elicited by changing the occlusal condition. It has to be pointed out, however, that given the fixed length of the read text and the relative small number of regressions performed during reading (Rayner, 2009; 10–15% of the time), the changes in saccade amplitude can be expected to be strongly (and negatively) correlated to those in saccade (fixation) frequency.

### 4.2 EMG asymmetry and oculomotor behaviour during a Reading task

Findings from this study suggest that oculomotor behaviour is modified by the occlusal condition.

In our study, during the second reading session, we did not observe an average decrease in the total reading time. Conversely, we noticed an increase of the frequency of fixations and a decrease in the average duration of fixations. This suggests that removing the imprint for the second reading session might have partially negated the typically positive impact that a second reading has on oculomotor metrics, consisting in a decrease in fixation frequency and duration. This implies that the influence of occlusal condition on reading performance may be significant enough to override the decrease in fixation frequency expected from the familiarity and repetition of the reading task. This hypothesis is supported by the finding that the increase in fixation frequency, induced by switching from corrected to the habitual condition, was positively correlated with the corresponding increase in EMG asymmetry during clenching. Since the duration of fixations was on the average reduced, as would have been expected during a second repetition of the reading task, it could be proposed that occlusal disharmony is hampering the sequence of eye movements more than the processing of visual information, which determines the duration of the fixation period. In some subjects, fixation duration showed little changes or a frank increase switching from corrected to habitual condition: consequently, they were reading faster with the imprint interposed between the arches. In this restricted population, the occlusal disharmony has not only increased the number of fixations but has also counterbalanced or overwhelmed the decrease in fixation duration expected by task repetition.

Beyond the changes in oculomotor metrics elicited by changing the occlusal condition, it has to be stressed that within our population oculomotor metrics in habitual occlusion are related to the trigeminal asymmetry: subjects with larger asymmetries show in fact higher number of fixations of shorter duration with respect to less asymmetric subjects. In other words, trigeminal asymmetry seems to determine the reading pattern of the individual subjects. This gives rise, within the population, to a trade-off between fixation duration and frequency, with an inverse relationship between the two oculomotor metrics. Higher number of fixations is indicative of a reduced saccadic amplitude and could be attributable, in principle, to a reduction in the span of text that can be processed by the reader, leading to a shift in reading dynamics/patterns characterized by an increased number of fixations (i.e., a reduced saccade amplitude) with shorter durations. Higher numbers of fixations and reduction of their duration with respect to normal controls has been also observed in offspring of late-onset Alzheimer’s disease (O-LOAD), as reported by Fernández et al. (2022). The authors concluded that “This eye movement pattern could be considered an early marker of oculomotor impairment.” They claimed that the control group processed and integrated the full meaning of a sentence through larger saccades and spending more time on the fixated words, whereas the O-LOAD group had difficulties in extracting information from the text, being constrained to a reading pattern characterized by shorter saccades and less time on the fixated word. In other words, the O-LOAD subjects could not analyse the same span of words as the control group.

The reading difficulties elicited by occlusal disharmony could be linked to a diminished capacity in processing visual input coming from the parafoveal region, which is crucial for efficient reading (Schotter et al., 2012): this would explain the higher fixation number and reduced fixation duration observed in our study. A parafoveal vision decline was observed in older readers (Payne and Federmeier, 2017). Indeed, these subjects showed a reduced ability to quickly combine the meanings of words seen in their peripheral and foveal vision (Payne and Federmeier, 2017). This could be related to neuroanatomical and functional changes induced by aging, particularly in the frontal cortex (Fabiani, 2012). Interestingly, in the presence of a trigeminal sensory-motor imbalance, a reduced performance was observed in the Spinnler-Tognoni test, consisting in the retrieval of target numbers within numerical matrices that must be scanned line by line (Spinnler and Tognoni, 1987). When the asymmetry was corrected by bite/oral prosthesis placement, it was observed an improvement in scanning velocity of the matrix, as well as in the speed of target retrieval, without any increase in error rate. These results contradicted the well-known trade-off between speed and accuracy (Fitts, 1954) and could be well explained by an increase in the subject’s ability to extract parafoveal information. This results in a higher number of items that can be processed simultaneously, allowing a faster execution of matrices scanning and targets retrieval. Based on these findings, the decrease in fixation duration following imprint removal could be regarded somehow differently than an improvement, in reading performance due to the repetition of the task. It could represent a change in reading pattern imposed by a reduced capability of processing visual information following the insurgence of a condition of occlusal disharmony. Further investigation is needed to clarify this issue.

### 4.3 Trigeminal and oculomotor pathways

Some of the central connections of trigeminal system may potentially account for an occlusal interference with oculomotor behaviour (Zhang et al., 2011; Liang et al., 2017). Indeed, trigeminal input reaches RF (Langer and Kaneko, 1990) and vestibular nuclear (Buisseret-Delmas et al., 1999) regions, as well as the nucleus prepositus hypoglossi (McCrea and Baker, 1985; Buisseret-Delmas et al., 1999) and the superior colliculus (Ndiaye et al., 2000), which have part in oculomotor control. These anatomo-functional relationships could account for pathological interference of mandibular movements with eye movements (Pratt et al., 1984; Zhang et al., 2011), as well as for the apparent interference existing between occlusal condition and ocular vergence (Marchili et al., 2016; Caruso et al., 2018). In principle, an asymmetry in the trigeminal input may complicate oculomotor control by affecting the activity of the structures mentioned above, resulting in the need of a larger number of saccades to read the same amount of text. Although we cannot completely reject this hypothesis, it must be pointed out that the observed changes in oculomotor behavior resembles to an adjustment in reading dynamics rather than to an oculomotor impairment.

Subjects with asymmetric sensorimotor trigeminal activity during clenching were characterized by anisocoria. Giving the established relationship between pupil size and LC activity, this finding suggests that in our sample the trigeminal asymmetry may lead to an asymmetry in the basal LC activity. The pathways linking proprioceptive trigeminal activity to LC activity have been recently reviewed (De Cicco et al., 2017). Fibres from the mesencephalic trigeminal nucleus reach the LC directly or through the reticular formation. In turn noradrenergic fibres from the LC contact mesencephalic trigeminal ganglion cells (Takahashi et al., 2010). Few LC axons enter the trigeminal motor nucleus where they enhance motoneuronal excitability (Samuels and Szabadi, 2008). These feedback projections may potentially enhance proprioceptive trigeminal and LC activity during chewing, giving rise to a progressive enhancement in cognitive performance (Tramonti Fantozzi et al., 2023). A predominant trigeminal sensorimotor activity on one side may therefore lead to an asymmetry in LC discharge, which can be further enhanced by the reciprocal LC connection between the two sides (Jones and Yang, 1985), which are inhibitory in nature (Cedarbaum and Aghajanian, 1977).

The condition could be detrimental for higher level sensory-motor and cognitive processes (De Cicco et al., 2014, 2016; Tramonti Fantozzi et al., 2019, 2021a,c), either by inducing an asymmetric body representation, or else an asymmetric hemispheric excitability, akin to the phenomenon described by Lomber and Payne (1996). The scanning dynamic of the text can be affected by such an asymmetry, contributing to the increased number of fixations (saccades) observed in the habitual occlusion condition compared to the occlusion corrected condition.

In conclusion, the influence of occlusion condition on oculomotor behaviour can be attributed to both the asymmetric trigeminal input to oculomotor structures and the effects of trigeminal asymmetries on higher visuo-spatial functions.

## Data Availability

The datasets presented in this study is freely available in an online repository at: https://osf.io/7xe5f/.
